# Induction of pan-azole resistance in *Cryptococcus neoformans* by agrochemical azole uniconazole through upregulation of efflux and chromosomal disomies

**DOI:** 10.3389/fcimb.2025.1664896

**Published:** 2025-09-08

**Authors:** Maoji Zhang, Weihua Ma, Jing Wang, Feng Yang, Jingjing Zhong, Yi Xu

**Affiliations:** ^1^ Department of Pharmacy, Jianyang People’s Hospital, Chengdu, China; ^2^ Jinzhou Medical University Graduate Training Base (The 960th Hospital of PLA), Jinan, China; ^3^ Laboratory Department of Zhoucun District Center for Disease Control and Prevention, Zibo, China; ^4^ Department of Pharmacy, Zibo Zhoucun People’s Hospital, Zibo, China; ^5^ Department of Pharmacy, Shanghai Tenth People’s Hospital, School of Medicine, Tongji University, Shanghai, China; ^6^ Department of Pharmacy, The 960th Hospital of PLA, Jinan, China

**Keywords:** *Cryptococcus neoformans*, agrochemical azoles, medical azoles, cross-resistance, aneuploidy, efflux, AFR1

## Abstract

*Cryptococcus neoformans* is a fungus naturally found in the environment, particularly in soil, bird droppings, and trees. Cryptococcosis, caused by *Cryptococcus* spp., primarily *C. neoformans* and *C. gattii*, poses a significant threat to human health. Agrochemicals are widely used worldwide, and most applied agrochemicals are dispersed into the environment, which can have direct and indirect effects on human health. This study investigates the impact of the plant growth regulator uniconazole (UCZ) on antifungal resistance in *C. neoformans* using the H99 laboratory strain. We found UCZ to have potent antifungal activity, and exposure to UCZ induced genomic alterations, resulting in cross-resistance to both agricultural and medical azoles. The adaptors showed altered gene expressions across the genome, including efflux genes, as well as increased efflux pump activity. Deletion and overexpression of *AFR1* demonstrated its role in mediating resistance to azoles, with unexpected effects on amphotericin B sensitivity. These findings underscore the significant impact of agricultural agrochemicals on antifungal resistance development and the importance of considering environmental exposures in resistance management strategies.

## Introduction

The global population of immunosuppressed individuals is on the rise annually, primarily driven by several key factors. The HIV/AIDS epidemic remains a significant contributor, alongside advancements in medical care such as organ transplantation and chemotherapy, which necessitate immunosuppressive therapy. Moreover, the aging demographic worldwide has led to a higher prevalence of age-related conditions and chronic diseases, further compromising immune function. Additionally, environmental factors like pollution, exposure to toxins, and the effects of climate change have emerged as contributors to immunosuppression (Winans et al., 2011; Imberti et al., 2025). Concomitantly, opportunistic fungal infections have become a significant global health threat. Some common opportunistic fungal infections include candidiasis, aspergillosis, cryptococcosis, and mucormycosis (Gnat et al., 2021). In 2022, World Health Organization (WHO) published the first-ever list of fungal “priority pathogens” – a catalogue of the 19 fungi that represent the greatest threat to public health. Among them, *Cryptococcus neoformans*, *Aspergillus fumigatus*, *Candida albicans* and *Candida auris* are categorized as the “critical priority” group (WHO, 2022).

*C. neoformans* is an obligate aerobe found worldwide. It thrives in natural environments such as pigeon droppings, soil contaminated with avian guano, and decaying tree barks (Sorrell and Ellis, 1997). Inhalation of desiccated yeasts or spores from the environment poses a risk for cryptococcosis if not efficiently eliminated by host immune defenses. In immunocompromised individuals, failure to effectively eliminate the fungus can lead to its dissemination to the central nervous system, culminating in life-threatening meningitis (Zhao et al., 2023). Cryptococcal meningitis (CM) is the primary cause of adult meningitis in regions grappling with a high prevalence of HIV, notably in sub-Saharan Africa. Approximately 15% of HIV-associated fatalities worldwide are linked to CM, with three-quarters of these occurrences concentrated in sub-Saharan Africa (Rajasingham et al., 2017).

At present, only three classes of drugs are employed in the treatment of cryptococcosis: azoles, polyenes and flucytosine. Azoles are widely preferred for treating cryptococcosis due to their favorable safety profile and relatively affordable cost. In resource-limited countries, azoles are often the only available antifungal drugs for the treatment of cryptococcosis. These azole drugs include fluconazole (FCZ), itraconazole (ICZ), voriconazole (VCZ) and posaconazole (PCZ). Due to its toxicity profile, use of polyenes, such as amphotericin B (AMB), is limited to more serious cases or when other treatment options have failed. Monotherapy with flucytosine, also known as 5-flucytosine (5FC), is not recommended due to rapid evolution of drug resistance. 5FC is typically used in combination with amphotericin B. However, its availability is limited in some regions, particularly in low-resource settings (Iyer et al., 2021).

Due to the limited antifungal arsenal, resistance is increasingly becoming a major obstacle in treatment of cryptococcosis. A recent assessment of drug resistance in *Cryptococcus* species unveiled that 10.6% of clinical isolates demonstrate resistance to FLC, with this proportion escalating to 24.1% in cases of disease relapse among patients (Bongomin et al., 2017). Resistance to azoles is generally due to altered target and increased efflux. Azoles target the synthesis of fungal-specific ergosterol by inhibiting the fungal cytochrome P450-dependent enzyme lanosterol 14-α-demethylase, which is encoded by *ERG11*. Point mutations and aneuploidy-mediated copy number increase represent the primary mechanisms governing the regulation of *ERG11* (Rodero et al., 2003; Sionov et al., 2010, 2012; Stone et al., 2019; Iyer et al., 2021). Efflux pumps, as membrane transport proteins, play an active role in expelling antifungal drugs from fungal cells, consequently lowering their intracellular levels and diminishing the efficacy of the drugs. These pumps have the capacity to confer resistance to various classes of antifungal agents and are frequently upregulated in response to drug exposure or genetic mutations. In *C. neoformans* genome, *AFR1*, *AFR2*, and *MDR1* encode ATP binding cassette transporters and Afr1 is the major efflux pump (Chang et al., 2018). Notably, both the *ERG11* and *AFR1* genes are located on chromosome 1 (Chr1) within the *C. neoformans* genome. Consequently, aneuploidy of Chr1 leads to the simultaneous upregulation of both genes, resulting in enhanced resistance to fluconazole. This mechanism of drug resistance occurs both *in vitro* and *in vivo* settings (Sionov et al., 2010; Stone et al., 2019; Yang et al., 2021a).

In addition to direct exposure to pharmaceutical agents, the presence of antifungal agents in environmental niches like soil and water can substantially augment the selection and propagation of resistant fungal strains. This phenomenon can arise from the agricultural application of antifungal agents, including fungicides used on crops, or from the discharge of antifungal agents into the environment through wastewater originating from hospitals or agricultural operations. Consequently, environmental reservoirs may act as focal points for the emergence and dissemination of antifungal resistance, thereby posing a dual risk to human health and agricultural sustainability (Bastos et al., 2021). The exposure of *Aspergillus* fungi to agricultural pesticides during field applications is increasingly recognized as a potential mechanism for acquiring mutations in the *CYP51* gene. Such mutations may confer cross-resistance to voriconazole, the primary treatment option for invasive aspergillosis (Le Pape et al., 2016; Zhou et al., 2021). The application of pesticides, notably azole fungicides, is escalating in sub-Saharan nations, particularly on cash crops, which represent significant economic assets for these regions (Snyder et al., 2015). Desiccated yeasts or spores of *C. neoformans* are quite likely to be present in crops that are attractive for avian fauna. Thus, the possibility of these yeasts acquiring resistance to medical azoles through exposure to agrochemical azoles in the field cannot be excluded.

Research suggests that exposure of *C. neoformans* to azole pesticides such as tebuconazole (TCZ) results in the upregulation of *ERG11* and *AFR1* expression, leading to cross-resistance to medical azoles (Bastos et al., 2018; Drakulovski et al., 2023). Another study revealed that exposure to the non-azole agrochemical pyraclostrobin induced cross-resistance to medical azoles in *C. gattii*, a close relative of *C. neoformans*, by upregulating the expression of efflux genes *AFR1* and *MDR1* (Bastos et al., 2019). However, the precise mechanisms through which agrochemicals regulate the expression of these genes associated with antifungal resistance require further investigation.

Uniconazole (UCZ) is extensively employed as an agrochemical, primarily functioning as a plant growth regulator rather than a fungicide. Its widespread application in crops such as rice, wheat, barley, and soybeans aims to modulate plant growth, enhance stress tolerance, and improve yield (Saito et al., 2006). Despite its non-fungicidal nature, its agricultural use may still influence fungal populations and their dynamics within crop ecosystems. However, there is still a deficiency in studies addressing this matter.

In this study, we observed that UCZ demonstrated robust antifungal efficacy against the *C. neoformans* laboratory strain H99—a widely adopted, genomically stable reference strain that facilitates standardized resistance comparisons, though its phenotypic homogeneity may not fully reflect clinical isolate diversity. Exposure to low UCZ concentrations induced the development of Chr6 disomy (Chr6x2), resulting in cross-resistance to agricultural azoles UCZ and TCZ, as well as medical azoles ICZ, VCZ, and PCZ but not FLC. Higher UCZ concentrations elicited a range of genomic alterations, including euploidy, Chr1x2 alone or in conjunction with Chr4 or/and Chr6 disomy, and more complex forms of aneuploidy involving disomies of multiple chromosomes. Isolates obtained from exposure to elevated UCZ concentrations generally displayed pan-resistance to azoles including agricultural azoles UCZ and TCZ, as well as medical azoles FLC, ICZ, VCZ, and PCZ, but showed heightened sensitivity to AMB. Aneuploid isolates also exhibited cross-resistance to 5FC. Our findings revealed that UCZ adaptors commonly exhibited altered expression of numerous genes across the genome, including *ERG11* and efflux genes *AFR1* and *MDR1*, as well as increased efflux of rhodamine 6G. Deletion and overexpression of *AFR1* resulted in decreased and increased resistance to both agricultural and medical azoles, respectively, but *AFR1* was not associated with 5FC resistance. Unexpectedly, *AFR1* deletion conferred resistance to AMB, while overexpression led to hypersensitivity. Finally, we demonstrated the essential role of *AFR1* in UCZ-induced Chr1x2 formation.

## Materials and methods

### Strains and growth conditions

*C. neoformans* lab strain H99 was used as the wild-type strain. Constructions of *AFR1* deletion strain and *AFR1* overexpression strain were described in (Zhang et al., 2024). Stock cultures were preserved in 25% (v/v) glycerol (prepared in distilled water) and maintained at -80°C. Cells were routinely grown in Yeast extract-Peptone-Dextrose (YPD)-media (1% [w/v] yeast extract, 2% [w/v] peptone and 2% [w/v] D-glucose) at 30°C in a shaking incubator at 150–200 rpm. Synthetic dextrose (SD) was prepared with YNB (0.67% [w/v] yeast nitrogen base w/o amino acid and ammonium sulfate) and 2% [w/v] glucose. For solid medium, 2% [w/v] agar was added. Drugs were dissolved in dimethyl sulfoxide (DMSO) and stored at -20°C.

### Growth curve

Strains were streaked from -80°C freezer to YPD agar plates. After incubation at 30°C for 72 h, colonies were suspended in YPD broth, and cell density was determined by using a hemocytometer. The culture was adjusted to 2.5x10^3^ cells/mL in YPD broth with or without test drugs. Growth was monitored in a 96 well plate. The plate was incubated at 30°C. OD_600_ was monitored in a Tecan plate reader (Infinite F200 PRO, Tecan, Switzerland) at 1 h time intervals for 72 h. Data are represented as the mean ± SD of three biological replicates.

### Spot assay

Cells were suspended in distilled water and adjusted to 1x10^7^ cells/mL. Three microliters of 10-fold serial dilutions were spotted on solid medium (YPD-agar or SD-agar) with or without (control) drugs. The plates were incubated at 30°C and photographed after 72 h.

### Obtaining adaptors using low amount of uniconazole

Strains were streaked from -80°C freezer to YPD-agar plates. After incubation at 30°C for 72 h, colonies were suspended in YPD broth, and cell density was adjusted to 2.5x10^3^ cells/mL. Two milliliters of the culture was supplemented with or without 0.25 µg/mL UCZ. After 72 h incubation with shaking at 30°C, the culture was washed and diluted with distilled water. Approximately 200 cells were spread on YPD-agar plates. The plates were incubated at 30°C for 72 h. Ninety-five colonies were randomly tested for resistance to 1 μg/mL UCZ.

### Obtaining adaptors using high amount of uniconazole

Cells were suspended in distilled water and adjusted to 1x10^7^ cells/mL. One hundred microliters of cell suspension were spread on YPD-agar plates supplemented with 1 and 2 μg/mL UCZ. The plates were incubated at 30°C for 5 days (H99 derivatives) or 7 days (*afr1 Δ* derivatives). Thirty adaptors from each plate were randomly chosen. For each adaptor, 4–6 colonies of similar size were selected and frozen in 1 mL of 25% glycerol at -80°C.

### Efflux of rhodamine 6G

Strains were grown on YPD plates for 72 hours. Colonies were collected and suspended in phosphate-buffered saline (PBS). Cell density was adjusted to 1.0x10^8^ cells/mL. The cultures were incubated at 30°C for 1 hour with shaking for deprivation of glucose. Rhodamine 6G (Sigma-Aldrich, St Louis, MO, USA) was then added at a final concentration of 10 μM. The cultures were incubated at 30°C for 30 min with shaking. After washing three times with cold sterile PBS, the cells were resuspended in PBS. Glucose at a final concentration of 20 μM was added. After incubation at 30°C for 0, 20, 40, and 60 min with shaking, the fluorescence of the supernatant was measured with Thermo Scientific Varioskan Flash using the SkanIt software (excitation wavelength at 527 nm and emission wavelength at 555 nm). The experiment was performed three times.

### Next generation sequencing

DNA extraction, library construction and sequencing were performed as described previously (Yang et al., 2021c). Data was visualized using Y_MAP_ (Abbey et al., 2014). Raw fastq files were uploaded to Y_MAP_ (version 1.0) (http://lovelace.cs.umn.edu/Ymap/) (Abbey et al., 2014). Read depth was plotted as a function of chromosome position using the *C. neoformans* H99 reference genome (https://www.ncbi.nlm.nih.gov/assembly/GCF_000149245.1/).

### RNA-Seq

Strains were streaked from -80°C freezer to YPD-agar plates. After incubation at 30°C for 72 h, several colonies of each strain were suspended in distilled water. Cells were diluted and approximately 1000 cells were spread on YPD-agar plates. The plates were incubated at 30°C for 72 h. Cells were collected by centrifugation and flash frozen in liquid nitrogen. Three biological repeats of each strain were performed.

Total RNA extraction and purification, library construction, and sequencing were performed as described previously (Sun et al., 2023). Three biological replicates were obtained for each strain. Differential gene expression profiling was carried out using DESeq2 (Love et al., 2014) with standard parameters. Genes with FDR (False Discovery Rate)-adjusted p value <0.05 and expression fold changes of more than 1.5 or less than 0.67 were considered differentially expressed.

### Statistical analysis

Significance analysis of differences between growth curves was performed using Tukey HSD (Honestly Significant Difference) test.

### Data availability

The sequencing data have been deposited in the ArrayExpress database at EMBL-EBI (www.ebi.ac.uk/arrayexpress) under the accession numbers E-MTAB-14024, E-MTAB-14025, E-MTAB-14026 (DNA-Seq) and E-MTAB–14094 (RNA-Seq).

## Results

### Uniconazole has potent antifungal activity against *C. neoformans*

**Figure 1:**
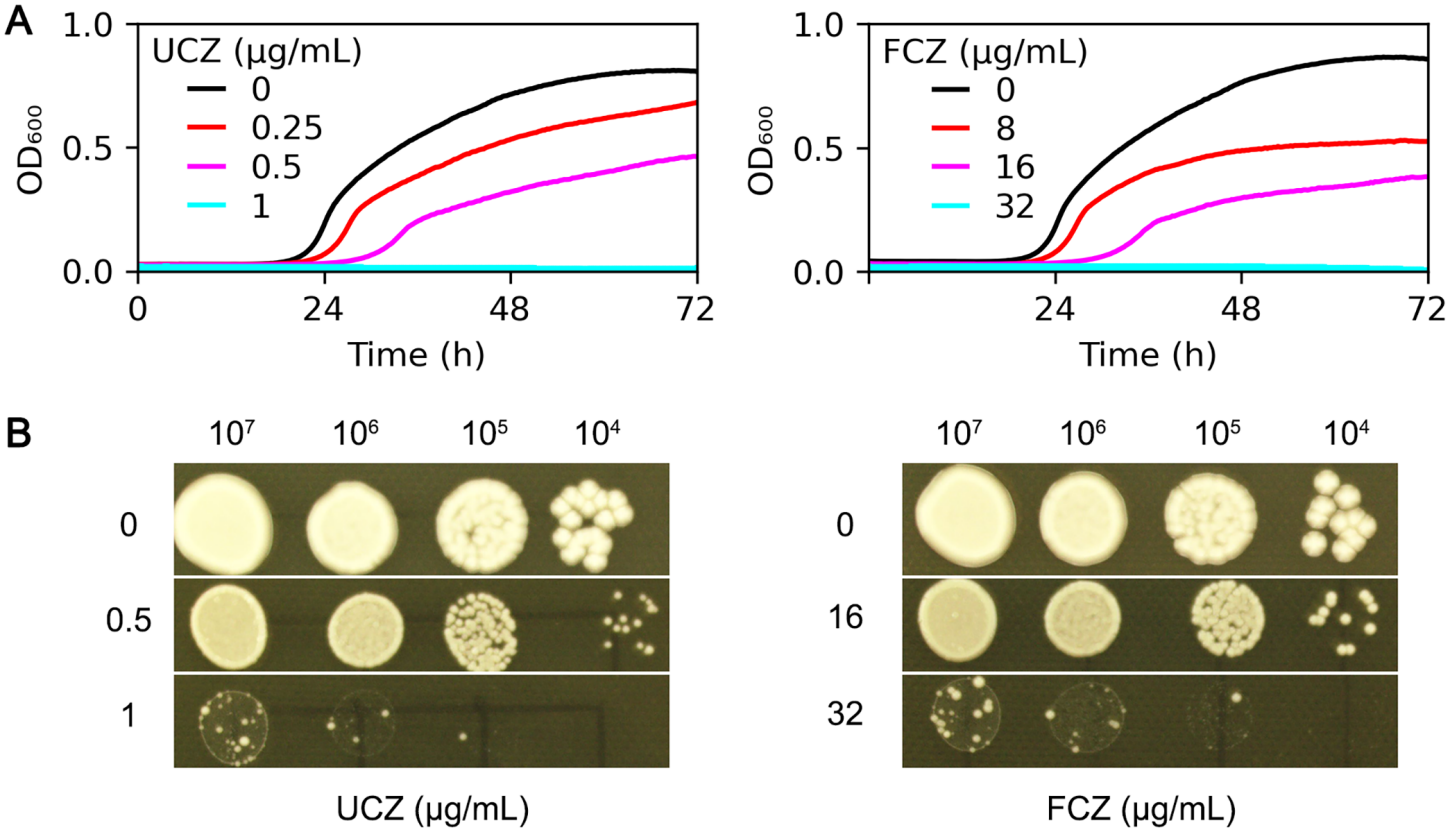
Evaluation of antifungal efficacy of uniconazole. **(A)** *C. neoformans* lab strain H99 was grown in YPD broth supplemented with uniconazole (UCZ, left panel) or fluconazole (FCZ, right panel). The growth was monitored for 72 h at a 1 h time interval. Shown is the mean ± SD of three biological replicates. **(B)** 3 μL of serial 10-fold dilutions of H99 cells were spotted onto YPD-agar plates containing the indicated drugs. The plates were incubated at 30°C for 72 h then photographed.

Antifungal efficacy of UCZ against *C. neoformans* lab strain H99 was evaluated using both broth medium and agar plates. In YPD broth, compared to the growth in the absence of UCZ, 0.25 and 0.5 μg/mL UCZ significantly (p<0.001, Tukey test) inhibited growth, and 1 μg/mL UCZ completely inhibited growth (**Figure 1A**, left panel). As a comparison, the medical azole FLC significantly inhibited growth of H99 at a concentration of 8 μg/mL significantly (p<0.001, Tukey test) inhibited growth of H99 (**Figure 1A**, right panel). On YPD-agar plates, UCZ and FLC at 1 μg/mL and 32 μg/mL, respectively, completely inhibited growth of H99 (**Figure 1B**). Thus, UCZ has potent inhibitory effect against H99.

### Exposure to sub-inhibitory concentration of uniconazole induces cross-resistance to agrochemical and medical azoles mediated by disomy of chromosome 6

**Figure 2:**
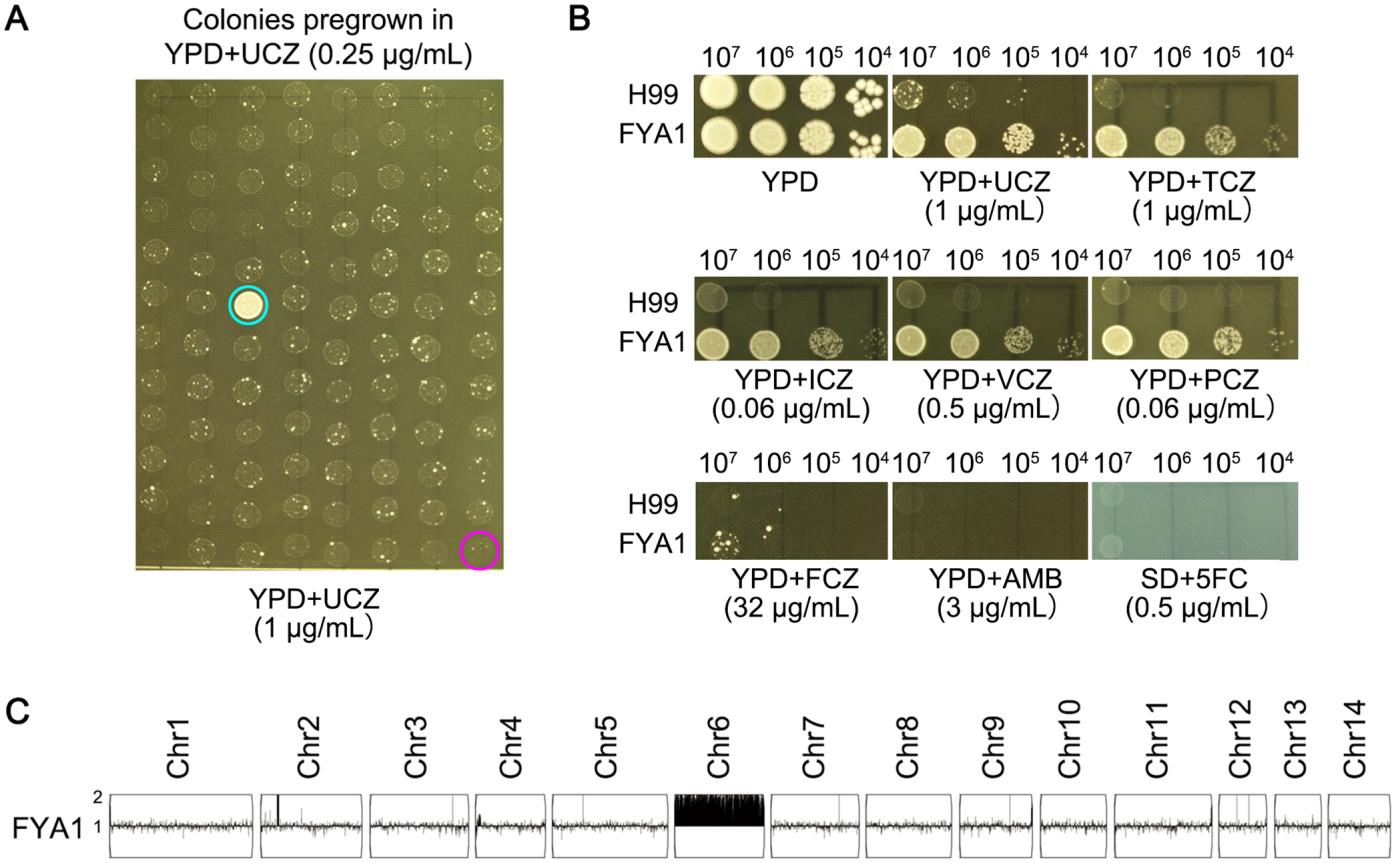
Low amount of uniconazole induces cross-resistance to agrochemical and medical azoles. **(A)** H99 was pre-grown in YPD broth supplemented with 0.25 μg/mL uniconazole (UCZ) for 72h. Ninety-five colonies were randomly isolated from the culture were compared to the parent for resistance to 1 μg/mL UCZ. Only adaptor (FYA1, cyan circle) could grow while the parent (magenta circle) could not. **(B)** Cross-resistance to agrochemical azoles and medical drugs was evaluated by spot assay. YPD-agar medium was used to test agrochemical azoles (UCZ and tebuconazole (TCZ)), medical azoles (fluconazole (FCZ), voriconazole (VCZ), posaconazole (PCZ) and itraconazole (ICZ)), and amphotericin B (AMB)). Synthetic dextrose (SD)-agar medium was used to test 5-flucytosine (5FC). The plates were incubated at 30°C for 3 days then photographed. **(C)** The adaptor was sequenced, and the karyotype was visualized using Y_MAP_. Read depth (normalized to that of the haploid parent) is shown on the y-axis on a log2 scale converted to absolute copy numbers.

We evaluated sub-MIC UCZ exposure for its potential to induce cross-resistance across agrochemical and medical drug classes. H99 was pre-grown in YPD broth supplemented with 0.25 μg/mL UCZ for 72 h. Ninety-five colonies were randomly tested for ability to grow in the presence of 1 μg/mL UCZ on YPD-agar plates. One colony (FYA1, indicated by cyan circle) could grow, while the rest 94 colonies as well as the parent (indicated by magenta circle) failed to grow (**Figure 2A**). Cross-resistance of the colony to other agrochemical azoles and medical antifungal drugs was assessed. Spot assay indicated the adaptor was resistant to both UCZ and TCZ, and it was resistant to medical azoles PCZ, VCZ and ICZ. However, it showed no change in susceptibility to FLC, AMB, or 5FC compared to the parent strain (**Figure 2B**). Whole genome sequencing indicated the adaptor had disomy of chromosome 6 (Chr6x2) (**Figure 2C**). Thus, exposure to low amount of UCZ induces cross-resistance to agrochemical azoles and some medical azoles, and the resistance is mediated by formation of Chr6x2.

### Inhibitory concentration of UCZ induces cross-resistance to agrochemical and medical azoles via formation of diverse aneuploidy

**Figure 3:**
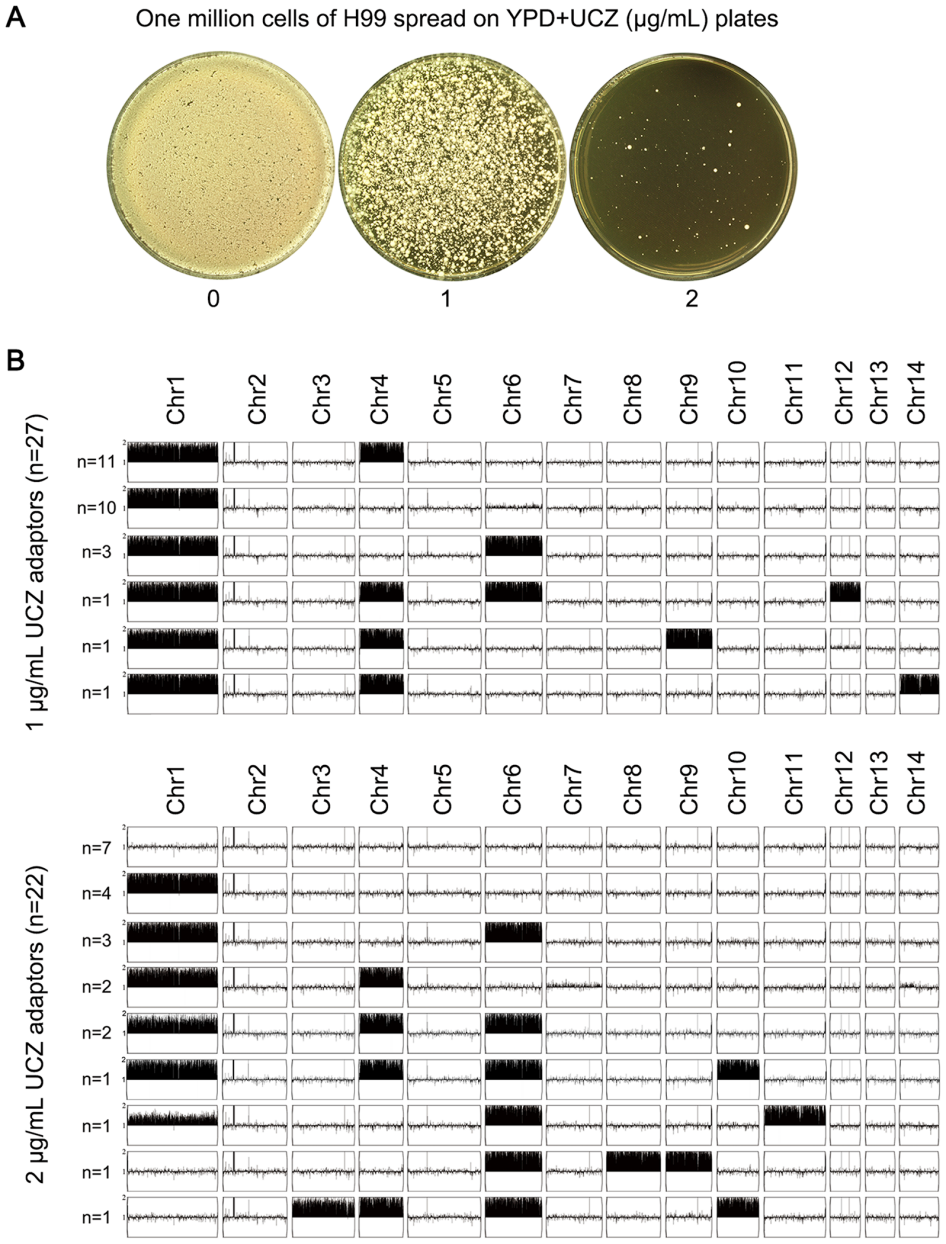
High amount of uniconazole selects adaptors with diverse aneuploidy. **(A)** One million cells of *C. neoformans* lab strain H99 were plated on YPD-agar plates supplemented with uniconazole (UCZ). The plates were incubated at 30°C for 5 days. **(B)** 27 and 22 UCZ-resistant colonies obtained from exposure to 1 and 2 μg/mL UCZ, respectively, were sequenced. The karyotypes were visualized using Y_MAP_.

Whether high-level UCZ exposure induces cross-resistance to medical antifungals was examined. Approximately one million cells of H99 were spread on YPD-agar plates supplemented with 1 and 2 μg/mL UCZ, respectively (**Figure 3A**). Thirty adaptors were randomly chosen from each drug plate. Spot assay indicated 27 and 21 adaptors chosen from 1 and 2 μg/mL UCZ plates, respectively, were resistant to UCZ (Data not shown).

All the 48 resistant adaptors were sequenced. Among the 27 adaptors isolated from 1 μg/mL UCZ plate, all had aneuploidy: 11 adaptors had disomy of Chr1 and Chr4 (Chrs(1,4)x2), 10 adaptors had Chr1x2 alone, 3 adaptors had Chrs(1,4,6)x2. 1 adaptor had Chrs(1,4,6,12)x2), 1 adaptor had Chrs(1,4,9)x2, and 1 adaptor had (Chr1,4,14)x2. Among the 21 adaptors isolated from 2 μg/mL UCZ plate, 7 adaptors were euploid and the rest 14 adaptors were aneuploid. Among the aneuploid adaptors, 4 adaptors had Chr1x2 alone, 3 adaptors had Chrs(1,6)x2, 2 adaptors had Chrs(1,4)x2, 2 adaptors had Chrs(1,4,6)x2, 1 adaptor had Chrs(1,6,11)x2, 1 adaptor had Chrs(6,8,9)x2, and 1 adaptor had Chrs(3,4,6,10)x2 (**Figure 3B**). Taken together, 41 out of 48 adaptors were aneuploid. Among them, 95.1% (39 out of 41) had disomy of Chr1 alone or together with disomy of other chromosomes, mainly Chr4 or Chr6.

**Figure 4:**
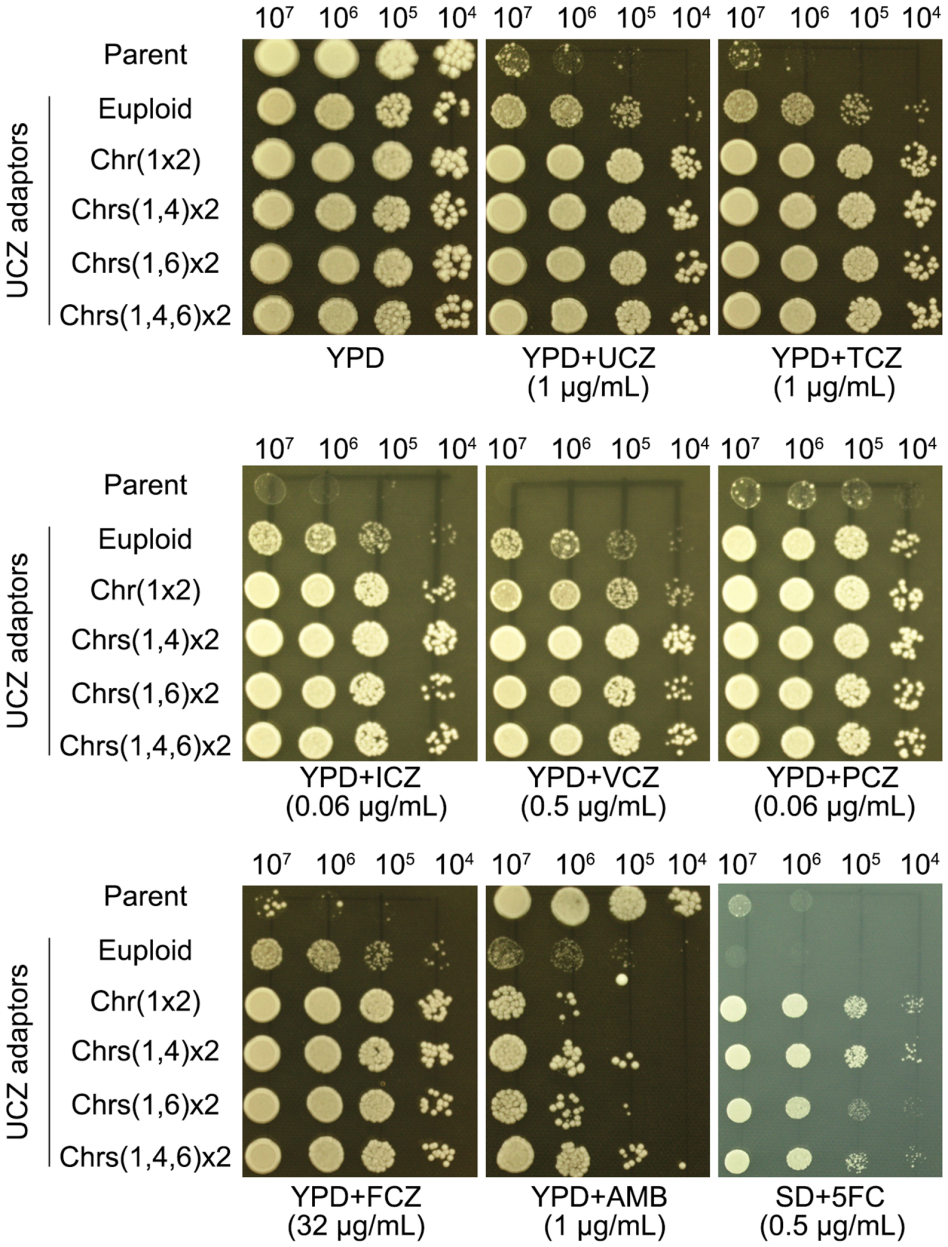
Evaluating susceptibility of uniconazole adaptors to agrochemical azoles and medical drugs. Adaptors with different karyotypes were tested for resistance to agrochemical azoles (UCZ and TCZ), medical azoles (FLC, ICZ, PCZ, VCZ), and other antifungal drugs (AMB and 5FC). The karyotypes are illustrated in the figure. For testing 5FC, SD-agar was employed, while YPD-agar was utilized for testing other drugs. Three microliters of cell suspensions, serially diluted tenfold, were spotted onto the plates. Subsequently, the plates were incubated at 30°C for 3 days before being photographed.

Six adaptors, each bearing a unique karyotype, were compared to their parent for resistance to drugs. All adaptors were more resistant than parent to both agrochemical azoles UCZ and TCZ, and medical azoles FLC, ICZ, PCZ and VCZ. And all adaptors were more susceptible to AMB. The euploid adaptor was not resistant to 5FC. All the aneuploid adaptors were resistant to 5FC (**Figure 4**).

Thus, adaptors isolated from high concentration UCZ plates were cross-resistant to agrochemical and medical azoles but more susceptible to AMB, and aneuploid adaptors were also resistant to 5FC.

### Aneuploidy leads to changes in gene expression across the genome

To investigate the impact of imbalanced chromosome copy number on gene expression across the whole genome, RNA sequencings (RNA-Seq) of UCZ-resistant adaptors bearing unique karyotypes were performed and compared to those of the parent. Relative expression values of each gene between adaptors and parent were calculated as a ratio, and the distributions of the ratios on each chromosome were plotted. Five aneuploid adaptors, bearing Chr6x2, Chr1x2, Chrs(1,4)x2, Chrs(1,6)x2), and Chrs(1,4,6)x2 respectively, and one euploid adaptors were tested. Among the aneuploids tested, Chr6x2 had 188 and 167 genes up and down regulated, respectively. Chr1x2 adaptor had 990 and 1040 genes up and down regulated, respectively. Chrs(1,4)x2 adaptor had 1245 and 1242 genes up and down regulated, respectively. Chrs(1,6)x2 adaptor had 1303 and 1402 genes up and down regulated, respectively. Chrs(1,4,6)x2 adaptor had 1624 and 1852 genes up and down regulated, respectively. Thus, Chr6x2 had the least number of differential genes. This might be because Chr6 (~0.79 Mb) was the smallest chromosome as compared to Chr1 (~1.43 Mb) and Chr4 (~0.94 Mb). Unexpectedly, the euploid adaptor also had a large number of differentially expressed genes: 1000 and 1400 genes were up and down regulated genes, respectively.

**Figure 5:**
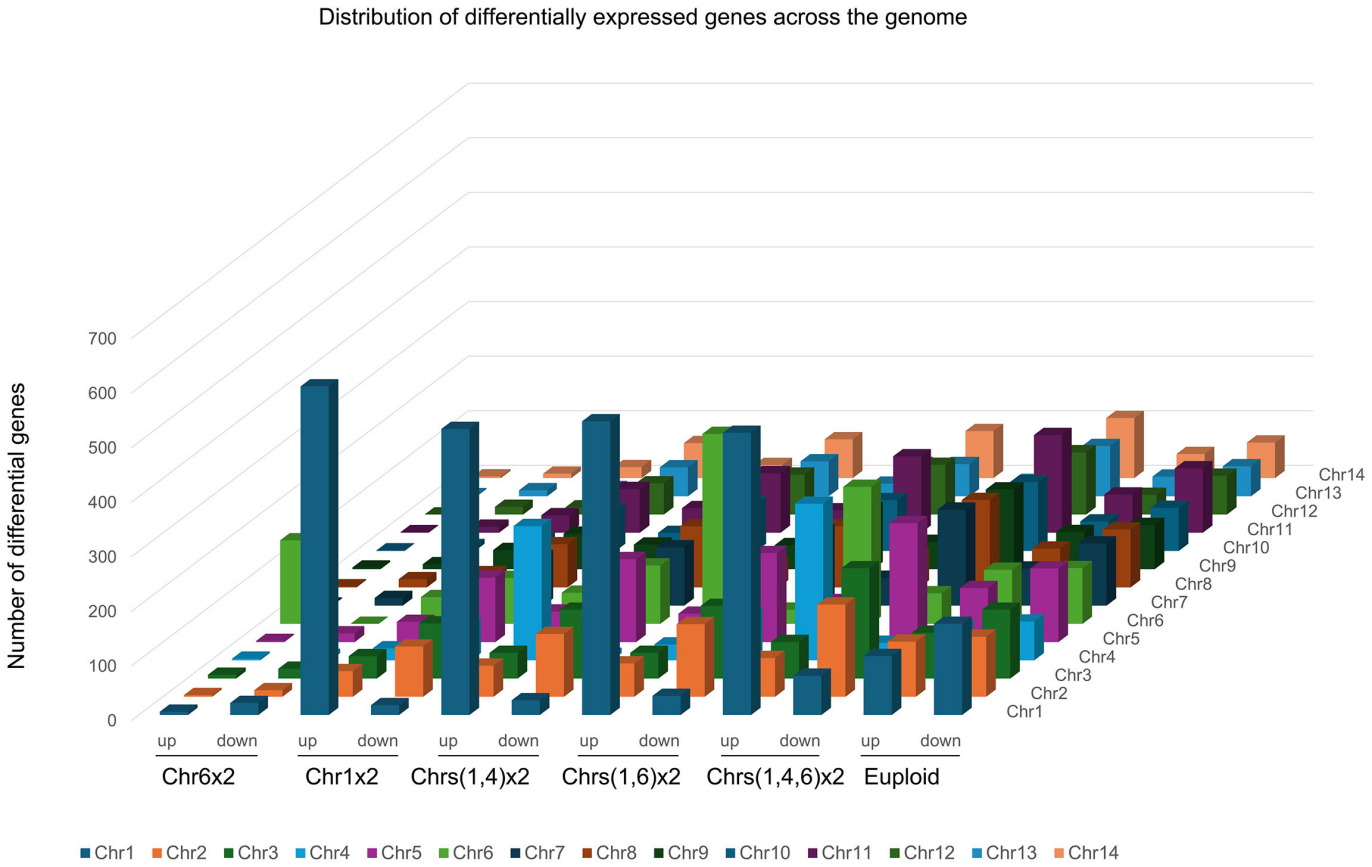
Impact of aneuploidy on gene expression across the genome. Transcriptomes of one euploid adaptor and 5 aneuploid adaptors, bearing Chr6x2, Chr1x2, Chrs(1,4)x2, Chrs(1,6)x2, and Chrs(1,4,6)x2, respectively, were compared to parent strain H99. Numbers of up (ratio>1.5, adjusted p-value < 0.05) and down (ratio<0.67, adjusted p-value < 0.05) regulated genes in each adaptor were plotted as a function of chromosome location.

In Chr6x2 adaptor, 81.4% (153 out of 188) of the up regulated and none of the 167 down regulated genes were on Chr6. In Chr1x2 adaptor, 60.8% (602 out of 990) and 1.7% (18 out of 1040) of the up and down regulated genes, respectively, were on Chr1. In Chrs(1,4) x2 adaptor, among the 1245 up regulated genes, 524 and 246 genes were on Chr1 and Chr4, respectively. Among the 1242 down regulated genes, 27 and 10 genes were on Chr1 and Chr4, respectively. Thus, 61.8% (770 out of 1245) and 3.5% (37 out of 1242) of the up and down regulated genes were on aneuploid chromosomes. In Chrs(1,6) x2 adaptor, among the 1323 up regulated genes, 538 and 348 genes were on Chr1 and Chr6, respectively. Among the 1402 down regulated genes, 35 and 26 genes were on Chr1 and Chr6, respectively. Thus, 67.0% (886 out of 1323) and 4.4% (61 out of 1402) of the up and down regulated genes were on aneuploid chromosomes. In Chrs(14,6) x2 adaptor, among the 1624 up regulated genes, 517, 287 and 251 genes were on Chr1, Chr4 and Chr6, respectively. Among the 1852 down regulated genes, 72, 32 and 56 genes were on Chr1 and Chr6, respectively. Thus, in aneuploid adaptors, 65.0% (1055/1624) of upregulated genes localized to aneuploid chromosomes, whereas downregulated genes were primarily on euploid chromosomes (8.6% aneuploid; 160/1852). In contrast, both up- and down-regulated genes were scattered genome-wide in the euploid adaptor (**Figure 5**).

### Uniconazole adaptors have increased expression of efflux genes and enhanced efflux pump activity

**Figure 6:**
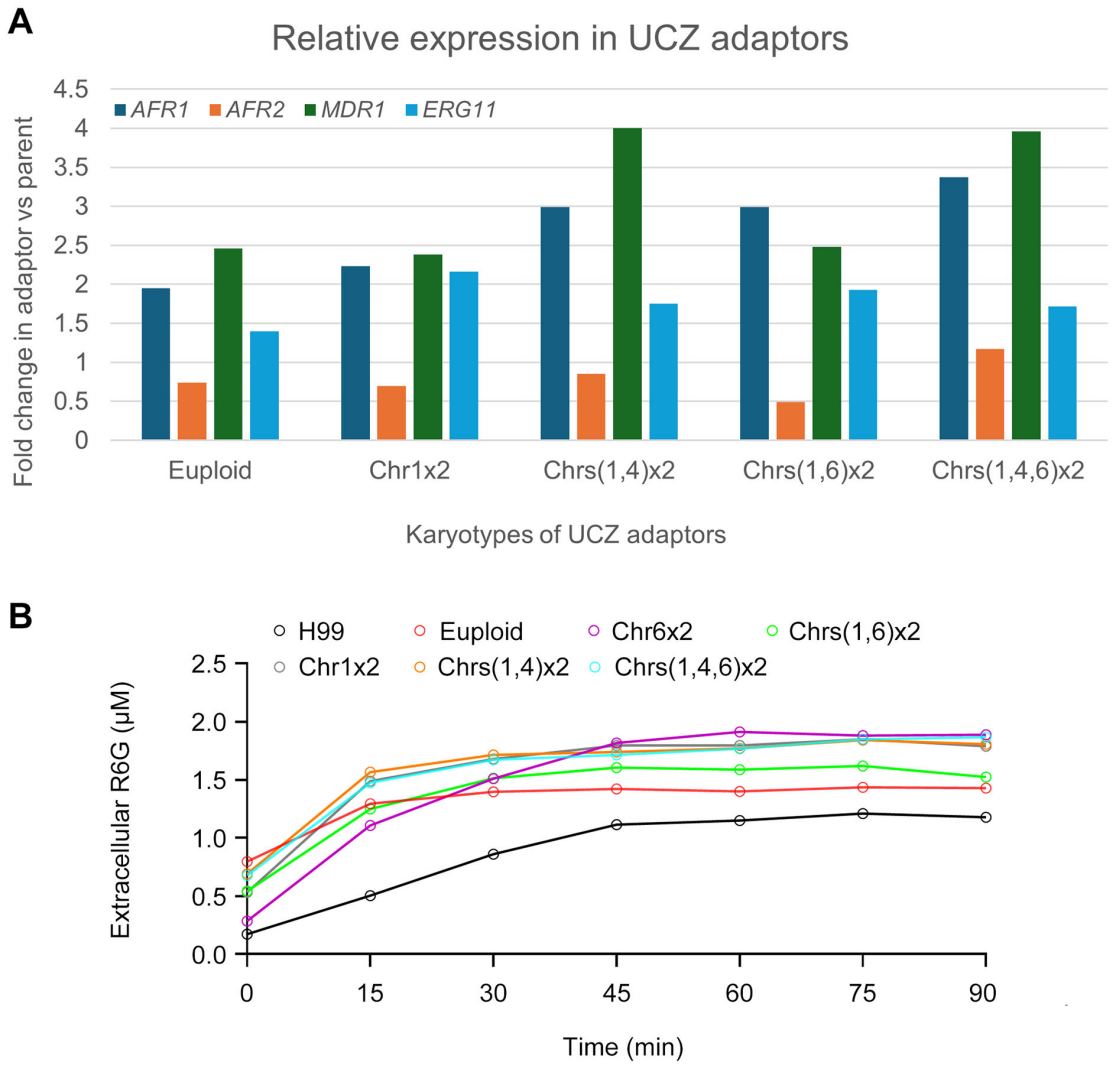
Expression of genes associated with azole resistance and efflux pump activity in uniconazole adaptors. **(A)** Relative expression of *ERG11*, *AFR1*, *AFR2*, and *MDR1* genes in uniconazole adaptors compared to H99, as determined by RNA-seq analysis. **(B)** Rhodamine 6G efflux assay in the presence of glucose. Fluorescence intensity was measured at an excitation wavelength of 527 nm and an emission wavelength of 555 nm. Data represent the mean ± SD of three biological replicates. Time points are indicated in the figure.

Resistance to azoles is generally due to altered target and increased efflux (Lee et al., 2023). In *C. neoformans* genome, *ERG11* (CNAG_00040) encodes the target protein of azoles. *AFR1* (CNAG_00730), *AFR2* (CNAG_00869), and *MDR1* (CNAG_00796) encode the major efflux pumps (Basso et al., 2015; Chang et al., 2018). We examined the expressions of *ERG11, AFR1, AFR2* and *MDR1* in adaptors. According to the RNA-Seq, in the euploid, Chr1x2, Chrs(1,4)x2, Chrs(1,6)x2 and Chrs(1,4,6)x2 adaptors, expressions of *ERG11*, *AFR1* and *MDR1* were significantly higher than parent (fold changes changes >1.5, adjusted p values<0.05) (**Figure 6A**).

To further investigate whether the activity of efflux pumps was involved, we performed a rhodamine 6G (R6G) efflux assay. In this assay, we measured extracellular concentrations of R6G in parent and UCZ adaptors over time in the presence of glucose. 6 adaptors were tested, bearing euploidy, Chr1x2, Chrs(1,4)x2, Chrs(1,6)x2, Chrs(1,4,6)x2 and Chr6x2, respectively (**Figure 6B**). All adaptors had significantly more extracellular R6G than parent (p<0.001, Student’s t-test).

Overall, these results suggest that in *C. neoformans*, the expression levels and activity of *ERG11*, *AFR1* and *MDR1* may be important factors for azoles resistance. Particularly, increased expression and activity of these efflux pumps contribute to pan-azole resistance in UCZ selected adaptors.

### *AFR1* is required for uniconazole induced formation of Chr1 disomy

**Figure 7:**
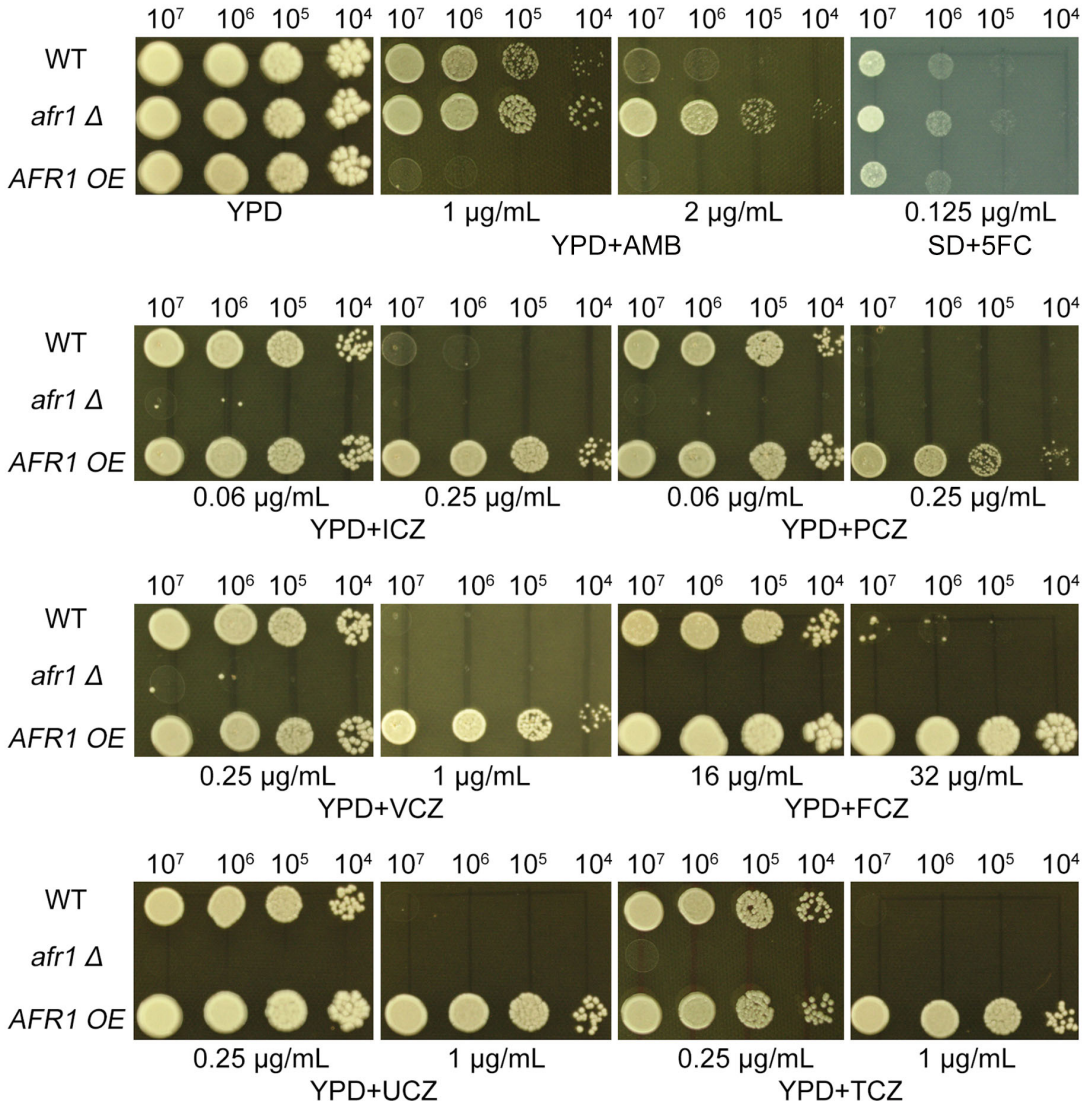
Impact of *AFR1* deletion and overexpression on susceptibility to drugs. The *AFR1* deletion (*afr1 Δ*) and overexpression (*AFR1* OE) strains were compared to the wild-type strain H99. SD medium was utilized for testing 5FC, while YPD medium was employed for testing other drugs. Drug concentrations are specified in the figure. The plates were incubated at 30°C for 3 days prior to being photographed.

To explore *AFR1*’s involvement in adapting to UCZ, we constructed strains with deleted and overexpressed *AFR1*. Spot assay indicated deletion of *AFR1* caused hypersensitivity to agrochemical azoles including UCZ and TCZ, and medical azoles including FCZ, ICZ, PCZ and VCZ. Consistently, overexpression of *AFR1* resulted in resistance to these azoles. Interestingly, deletion and overexpression of *AFR1* conferred resistance and hypersensitivity to AMB, respectively. Inconsistent with previous report (Chang et al., 2021), here we found deletion and overexpression of *AFR1* did not alter susceptibility to 5FC (**Figure 7**).

**Figure 8:**
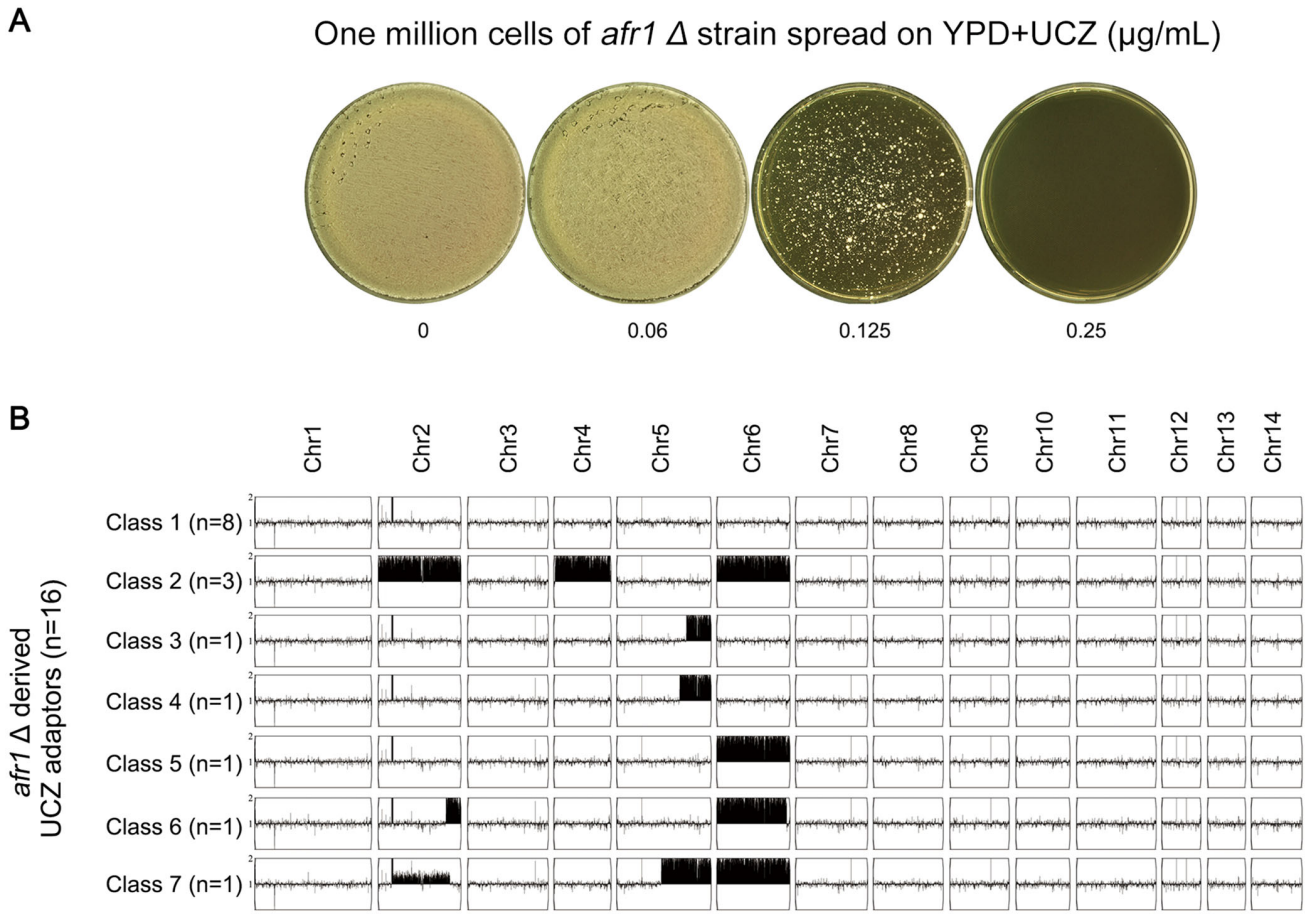
Requirement of *AFR1* for uniconazole induced Chr1 disomy formation. **(A)** One million cells of *AFR1* deletion strain were spread on YPD-agar plates supplemented with uniconazole (UCZ). The plates were then incubated at 30°C for 7 days. Thirty adaptors were randomly selected from the plate with a UCZ concentration of 0.125 μg/mL. **(B)** Sixteen UCZ-resistant adaptors were sequenced, and their karyotypes were visualized using Y_MAP_. The adaptors were classified into 7 categories based on their karyotypes. The figure illustrates the number of adaptors belonging to each category and their respective karyotypes.

Next, we explored how *AFR1* deletion strain adapted to UCZ. The deletion strain was spread on YPD-agar plate supplemented with 0.125 μg/ml UCZ (**Figure 8A**). Thirty adaptors were randomly tested. Only 16 adaptors were more resistant to UCZ (data not shown). Whole genome sequencing indicated none of the 16 resistant adaptors had Chr1x2. Eight adaptors were euploid, and the rest 8 adaptors were aneuploid: 3 adaptors had Chrs(2,4,6)x2. 2 adaptors had segmental disomy of Chr5 from ~1.24 Mb and ~1.37 Mb to the right telomere, respectively. 1 adaptor had Chr6x2. 1 adaptor had Chr6x2 as well as segmental disomy of Chr2 from ~1.35 Mb to the right telomere. 1 adaptor had Chr6x2 and segmental disomy of Chr2 from ~ 0.30 Mb to the right telomere, and segmental disomy of Chr5 from ~ 0.88 Mb to the right telomere (**Figure 8B**). Thus, *AFR1* was required for UCZ induced formation of Chr1x2.

## Discussion

In our investigation of the impact of the agrochemical azole UCZ on *C. neoformans* populations, we focused on genome instability, particularly on the occurrence of aneuploidies, and the development of pan-azole resistance. Our findings reveal that exposure to UCZ results in elevated rates of chromosome missegregation, leading to the emergence of various aneuploid adaptors within the population. The duplication of Chr1 (Chr1x2), either alone or in conjunction with disomy of other chromosome(s), emerged as the predominant genomic alteration. We demonstrated that UCZ-induced adaptors generally exhibit heightened expression of efflux genes and increased efflux activity, contributing significantly to pan-azole resistance. Furthermore, our research highlighted the role of *AFR1*, an efflux gene located on Chr1, in conferring resistance to azoles and hypersensitivity to AMB, and its necessity for UCZ-induced Chr1x2 formation. While this aneuploidy-based mechanism significantly elevates expression of resistance genes (*ERG11*, *AFR1*, *MDR1*), we acknowledge potential contributions from complementary pathways, including regulatory mutations in transcription factors, non-chromosomal resistance mechanisms (e.g., efflux pump epigenetics), or synergistic interactions with point mutations. Given the reliance on the reference strain H99 in this study, validating these findings across diverse *C. neoformans* genetic backgrounds represents a critical next step (Kassaza et al., 2022) for establishing broader ecological and clinical relevance.

The observed AMB resistance in *AFR1*-deleted strains—contrasted with hypersensitivity in overexpressors—suggests complex roles for this efflux pump beyond azole transport. We propose two non-exclusive mechanisms: First, *AFR1* deletion may induce compensatory membrane remodeling (e.g., reduced ergosterol or altered lipid saturation), limiting AMB’s pore-forming capacity by altering target availability. Second, loss of *AFR1* could dysregulate ion homeostasis (particularly mitochondrial Ca²^+^ flux), inadvertently activating stress responses that fortify membrane integrity. Conversely, *AFR1* overexpression likely imposes dual vulnerabilities: metabolic drain from hyperactive efflux depletes ATP reserves needed for membrane repair, while disrupted phospholipid asymmetry exposes inner membrane lipids, enhancing AMB binding. This inverse phenotype pattern implies that efflux pumps critically modulate membrane architecture in ways that unexpectedly dictate polyene susceptibility.

Previously, we observed that exposure to even mild stresses, such as FLC and the endoplasmic reticulum stressor tunicamycin, were capable of inducing aneuploidy formation in both *C. neoformans* (Yang et al., 2021a) and *Candida albicans* (Yang et al., 2021b, 2023). The current study underscores that even low doses of UCZ are sufficient to induce aneuploidy formation, which in turn leads to pan-azole resistance. Together, these studies indicate that genome instability in fungi, including *C. neoformans*, can be easily induced by even weak stress. Thus, although the levels of fungicides in the environment might be low, the induction of mutations and subsequent selection of resistant isolates can still occur in *C. neoformans*. Our study highlights the potential for widespread resistance development in environments where azoles are routinely used, such as agricultural settings. Similarly, in bacterial studies, sub-inhibitory concentrations, which are much lower than MIC, of antibiotics have been shown to promote the selection of cross-resistance to clinically significant antibiotics (Stanton et al., 2020; Sanz-Garcia et al., 2022). This raises concerns about the potential for antibiotics present in natural environments to drive the evolution of multidrug-resistant bacteria.

Another intriguing aspect revealed in this study is the diversity of genomic changes observed within the population. It’s notable that both low and high doses of UCZ led to the selection of different aneuploid chromosomes, suggesting a nuanced response to the drug’s concentration gradient. Furthermore, adaptors on the same drug plate exhibited a wide array of genomic alterations, underscoring the dynamic nature of the adaptive process. This diversity mirrors our previous findings with FLC, where adaptors on the same drug plate in *C. albicans* displayed varied karyotype changes (Yang et al., 2023). Aneuploidy, by its nature, alters the copy number of hundreds of genes on the affected chromosome, resulting in transcriptional and translational changes across the genome (Pavelka et al., 2010). In our study, both euploid and aneuploid adaptors showed altered gene expressions, indicating widespread genomic effects of UCZ exposure. This broad spectrum of genomic changes suggests that UCZ-selected adaptors have the potential to acquire pleiotropic traits. For instance, they may develop cross-resistance to both agricultural and medical azoles while simultaneously exhibiting hypersensitivity to AMB, another class of antifungal agent. This multifaceted adaptation underscores the complexity of fungal responses to environmental pressures and emphasizes the need for comprehensive strategies to manage antifungal resistance.

In conclusion, our study unveils the profound impact of agrochemicals, such as UCZ, on the development of antifungal resistance in *C. neoformans*. Exposure to UCZ induced genomic alterations that conferred cross-resistance to multiple agricultural and medical azoles, highlighting the significant role of agricultural practices in shaping antifungal resistance profiles. These findings underscore the necessity of considering environmental exposure to agrochemicals in the context of antifungal resistance surveillance and management strategies. Moreover, our study sheds light on the intricate interplay between agrochemical azoles, genome stability, and antifungal resistance in *C. neoformans*. By elucidating these mechanisms, we pave the way for developing targeted strategies to mitigate the emergence and spread of resistant fungal strains, thereby safeguarding both agricultural and clinical domains against the threat of fungal infections.

## Data Availability

The datasets presented in this study can be found in online repositories. The names of the repository/repositories and accession number(s) can be found in the article/Supplementary Material.
